# WSNEAP: An Efficient Authentication Protocol for IIoT-Oriented Wireless Sensor Networks

**DOI:** 10.3390/s22197413

**Published:** 2022-09-29

**Authors:** Fumin Yi, Lei Zhang, Lijuan Xu, Shumian Yang, Yanrong Lu, Dawei Zhao

**Affiliations:** 1 Shandong Provincial Key Laboratory of Computer Networks, Shandong Computer Science Center (National Supercomputer Center in Jinan), Qilu University of Technology (Shandong Academy of Sciences), Jinan 250014, China; 2 School of Safety Science and Engineering, Civil Aviation University of China, Tianjin 300300, China

**Keywords:** IIoT, authentication, efficient, PUF, wireless sensor network

## Abstract

With the development of the Industrial Internet of Things (IIoT), industrial wireless sensors need to upload the collected private data to the cloud servers, resulting in a large amount of private data being exposed on the Internet. Private data are vulnerable to hacking. Many complex wireless-sensor-authentication protocols have been proposed. In this paper, we proposed an efficient authentication protocol for IIoT-oriented wireless sensor networks. The protocol introduces the PUF chip, and uses the Bloom filter to save and query the challenge–response pairs generated by the PUF chip. It ensures the security of the physical layer of the device and reduces the computing cost and communication cost of the wireless sensor side. The protocol introduces a pre-authentication mechanism to achieve continuous authentication between the gateway and the cloud server. The overall computational cost of the protocol is reduced. Formal security analysis and informal security analysis proved that our proposed protocol has more security features. We implemented various security primitives using the MIRACL cryptographic library and GMP large number library. Our proposed protocol was compared in-depth with related work. Detailed experiments show that our proposed protocol significantly reduces the computational cost and communication cost on the wireless sensor side and the overall computational cost of the protocol.

## 1. Introduction

The Industrial Internet of Things (IIoT) is an application of the Internet of Things in the industrial field [1,2,3,4]. IIoT achieves the purpose of improving factory manufacturing efficiency and reducing product production costs through technologies such as sensors, controllers, mobile communication, and cloud computing [5,6,7,8,9]. The benefits that IIoT can bring are significant. In the field of aviation, an airline can save 1% of fuel through IIoT, and airlines save $30 billion a year. In the field of power stations, the power station can save 1% of fuel through IIoT, and the power station can save $66 billion in operating costs [10,11]. Therefore, IIoT is gaining popularity in oil and gas, energy production, coal mining, chemical plants, automobile production, logistics processes, pharmaceutical plants, ship handling, and aviation operations [12,13,14].

IIoT has the characteristics of automation, intelligent interconnection, real-time monitoring, and collaborative control. IIoT can obtain a large number of important process parameters by deploying wireless sensors in a large number of key positions [15]. These important process parameters cannot be obtained by traditional industrial production lines. The factory uploads a large number of manufacturing process parameters to the cloud server. The cloud server feeds back the analysis results to the device through big data analysis technology. This model can optimize industrial production management and improve industrial production efficiency [16]. In this process, a large amount of private data collected by wireless sensors will be exposed on the Internet, which is easy to be stolen and attacked by hackers [17]. Therefore, identity authentication and key negotiation are very necessary for wireless sensor networks for IIoT. Many complex wireless-sensor-authentication protocols have been proposed. However, these complex protocols are computationally expensive for wireless sensors. We considered fine-grained reduction of the computational cost of wireless sensors from the protocol level. Thus, we proposed an energy-saving authentication protocol for IIoT-oriented wireless sensor networks. The main contributions of this paper are as follows:

### 1.1. Contribution

- The PUF chip is introduced in the protocol, and the challenge–response pairs generated by the PUF chip are saved and queried using Bloom filters. On the premise of ensuring the security of the physical layer of the device, the protocol reduces the computational cost of the wireless sensor side. The protocol introduces a pre-authentication mechanism, and the gateway and the cloud server are continuously authenticated. The pre-authentication mechanism reduces the overall computational cost of the protocol and improves the network communication model. Based on the same communication model, the introduction of a pre-authentication mechanism can reduce the number of communications.
- We carried out informal security analysis and formal security analysis of our proposed protocol through the Automated Validation of Internet Security-Sensitive Protocols and Applications (AVISPA) tool. The results, when compared with related work, show that our proposed protocol has more security properties.
- We implemented various security primitives using the MIRACL cryptographic library and GMP large number library. Our proposed protocol makes an in-depth comparison with related work. Detailed experiments show that our proposed protocol significantly reduces the computational cost and communication cost on the wireless sensor side and the overall computational cost of the protocol.

### 1.2. Paper Organization

The structure of the remaining sections is organized as follows. In Section 2, we review some previous research work in the area of authentication protocols. Section 3 describes the preparatory knowledge, assumptions, and symbol explanations for the paper. Section 4 describes the specific design implementation of the protocol. In Section 5, we perform an informal security analysis of the protocol and a formal security verification using the AVISPA tool. In Section 6, we compare our protocol with other related protocols in terms of security features, total computational cost, and wireless-sensor-side computational cost. Finally, we present the concluded and future works of this article in Section 7.

## 2. Related Works

Wireless sensor networks are widely used in the Industrial Internet of Things. In addition, wireless sensor networks are also very important in other fields. Therefore, many experts and scholars have proposed many authentication protocols for wireless sensor networks. Here, we provide a brief review of wireless-sensor-network authentication protocols.

Xiong Li et al. proposed a robust ECC-based privacy-preserving security authentication protocol for IIoT. The protocol introduces a fuzzy extractor to extract the user’s biometric information. The biometric information is combined with the user’s password to provide authentication for the user. It uses an elliptic curve cryptographic algorithm to provide security. However, the protocol has high computational and communication costs, so it does not meet the need for low energy consumption for wireless sensors [18]. A three-factor wireless-sensor-network user authentication protocol was proposed by Xiong Li et al. The protocol triple factor includes biometrics, user passwords, and smart cards. It implements richer physical characteristics. However, due to the use of an elliptic curve cryptographic algorithm, the efficiency of the protocol needs to be improved [19]. Joonyoung Lee et al. proposed a three-factor authentication protocol for wireless sensor networks based on honey lists. The protocol introduces honey-list technology to defend against smart card loss and offline guessing attacks. The protocol takes into account the limited performance of sensors, so it only uses hash functions and XOR operations. The protocol is efficient but does not prevent physical capture attacks on wireless sensors [20]. Considering the physical security of medical IoT devices, Tejasvi Alladi et al. proposed a three-entity medical IoT two-way authentication protocol. The protocol uses a PUF to ensure the physical security of the device. It uses a three-entity model of the sensor node, gateway, and cloud server. Additionally, it implements two-stage key negotiation. However, the protocol increases the wireless sensor energy consumption [21]. Fan Wu et al. proposed a new three-factor authentication protocol. Compared with some similar protocols, this protocol has better security and applicability. However, the protocol uses timestamps to resist replay attacks. It also cannot guarantee the physical security of wireless sensors [22]. Weizheng Wang et al. proposed a lightweight authentication protocol for wireless medical sensor networks based on blockchain and PUFs. This protocol solves the problems of physical layer security and excessive server concentration in wireless sensor networks [23]. Muhammad Tanveer et al. proposed an efficient authentication protocol for the Industrial Internet of Things. The protocol adopts Lightweight Authenticated Encryption (LWC), which improves the efficiency of the protocol. However, the protocol does not take into account the physical-layer security of the device [24]. Amir Masoud Aminian Modarres et al. proposed an improved lightweight two-factor authentication protocol for IoT applications. The protocol analyzes the weaknesses and vulnerabilities of other protocols and uses BAN logic to analyze the security of the protocol. The security and efficiency of the protocol are improved [25]. Sungjin Yu et al. proposed a robust authentication protocol for wireless medical sensor networks. The protocol adopts blockchain and physical unclonable functions, which solves the problems of over-centralization and physical-layer security. However, this greatly increases the computational cost of the protocol, especially the energy consumption on the wireless sensor side [26].

In addition to the wireless-sensor-network authentication protocol, Alireza Esfahani et al. designed a lightweight authentication protocol for machine-to-machine communication. The protocol uses only hash functions and XOR operations. It has small computational and communication costs and implements rich security features [27]. Hien-Ming Chen et al. proposed a security authentication protocol for the Internet of Vehicles. The protocol uses an XOR operation and hash function. It addresses offline identity guessing attacks, location spoofing, and replay attacks. It has good performance in terms of security and efficiency [28]. Soumya Banerjee et al. designed a lightweight, anonymous user authentication protocol for IoT environments. The protocol protects the physical integrity of the device by introducing a physically unclonable function [29]. Karanjeet Choudhary et al. designed an authentication protocol for communication between users and gateways by using hash functions, XOR operations, and symmetric encryption algorithms. The protocol is efficient and robust [30]. Aiming at the identity authentication problem in smart grids, Weizheng Wang et al. proposed a smart meter authentication protocol based on blockchain and an elliptic curve cryptographic algorithm. Compared with other smart meter authentication protocols, the protocol has improved security and performance [31].

These protocols adopted different security primitives and network models. Unlike these, we measured the energy consumption of wireless sensors in a fine-grained way at the protocol level. We reduced wireless sensor energy consumption by reducing wireless sensor computation. We improved the network communication model with a pre-authentication mechanism to reduce the number of communication interactions.

## 3. Preliminaries

This section describes the preparatory knowledge and preliminary information for the paper: a physically unclonable function, the Bloom filter, adversary models, network communication models, assumptions, and the notation used in the paper.

### 3.1. Physically Unclonable Function

A PUF is a hardware security primitive [32]. It is an irreversible random map based on physical disorder [33]. It generates a large number of challenge–response pairs using randomly varying parameters during chip fabrication. Due to the physical changes that occur naturally in semiconductor devices during the fabrication of the same wafer, the PUFs produced by different chips are different. It is difficult to physically replicate a PUF chip that produces the same challenge–response pair. PUFs can generate a large number of challenge–response pairs. At the same time, a PUF has two very important properties. First, a PUF can solve the key storage problem well, as the PUF can calculate the response value at any time through the challenge value. Second, a PUF has the ability to resist tampering, as any active manipulation of the PUF’s internal circuitry will disrupt the PUF’s challenge–response mechanism. Combining the two characteristics of the above PUF, the PUF chip can effectively resist reverse attacks, detection attacks, and fault-injection attacks against the physical security of the device [34,35,36,37].

A PUF works by implementing a challenge–response mechanism. With the same PUF, different inputs (challenge values) will produce different outputs (responses). The same input produces different outputs for different PUFs. We can describe it with the following equation, where $C_{i}$ represents the input and $R_{i}$ represents the corresponding output.

$$R_{i}=PUF_{i}\left(C_{i}\right)$$

### 3.2. Bloom Filter

Bloom filters are simple space- and time-efficient random data structures. One is used to represent a collection to support membership queries [38]. The Bloom filter consists of an *m* length array of bits and k hash functions. The *m* length array of bits is initialized with each bit set to 0. When a key is added to the set, *k* hash values are calculated with *k* hash functions, and the corresponding position in the array is 1. Finally, we determine whether the key is in the set by querying the corresponding bit value in the bit array. The Bloom filter has huge advantages in space and time but has the problem of miscalculation rate and element removal. Reference [39] introduced several Bloom filter variants, which reduce the miscalculation rate and solve the problem of element deletion. Reference [40] proposes a Bloom filter that associates a value with each element that has been inserted and implements an associative array map.

### 3.3. Communication Network Model

**Figure 1:**
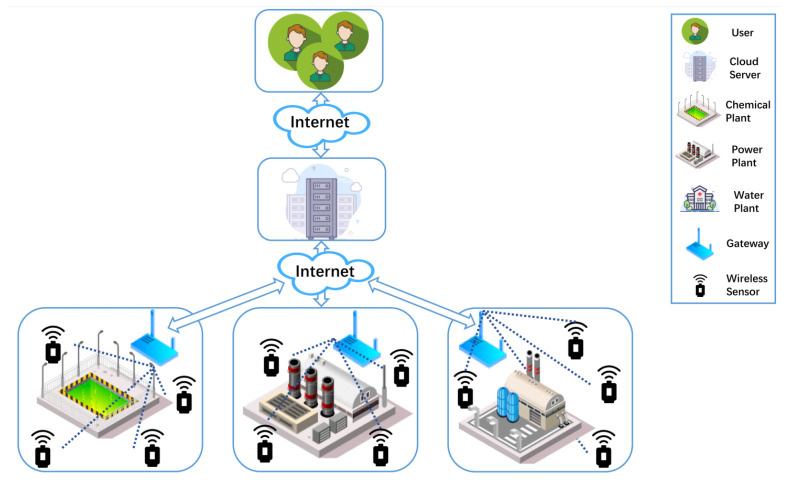
IIoT-oriented wireless sensor communication network model.

Figure 1 shows the IIoT-oriented wireless-sensor-communication network model. The model is divided into a three-tier infrastructure of wireless sensors, gateways, and cloud servers. After the wireless sensor is awakened, it collects real-time data about the environment or device. The wireless sensor sends the data to the gateway and then immediately goes into hibernation mode to save power. After receiving real-time data from numerous wireless sensors, the gateway uploads these real-time data to the cloud server.

### 3.4. Adversary Model

The paper uses the Dolev–Yao model, which is widely accepted by scholars. The model assumes that the attacker can control the entire network.

- The attacker can forge, eavesdrop, tamper, and replay communication information between the wireless sensor and gateway, and the gateway and cloud server.
- The attacker can intercept and store the messages sent by both sides of the communication.
- The attacker can participate in the operation of the protocol as a legitimate entity.

In addition, the attacker can capture expired session keys. It also can extract confidential information stored by the device through physical means [41].

### 3.5. Assumption

The following assumptions are made in this paper.

- Based on the actual IIoT application scenario, we assume that the cloud server is the only trusted institution to store confidential information. In this communication network model, only the cloud server is subject to advanced protection means such as physical isolation and professional security maintenance team. We treat the gateway as an untrusted party for two main reasons. Firstly, application software is installed in the gateway, and there may be loopholes in these application software. Secondly, the gateway may not have professional maintenance personnel, or the level of professional maintenance personnel may be insufficient.
- Each wireless sensor and gateway has its own PUF chip.
- The process of sending and receiving data in the registration phase is strictly protected. The attacker cannot obtain confidential information from the registration phase and cannot impersonate a legitimate device to register.

### 3.6. Symbol

Table 1 explains the meanings of the symbols used in this paper.

## 4. Protocol Design

**Figure 2:**
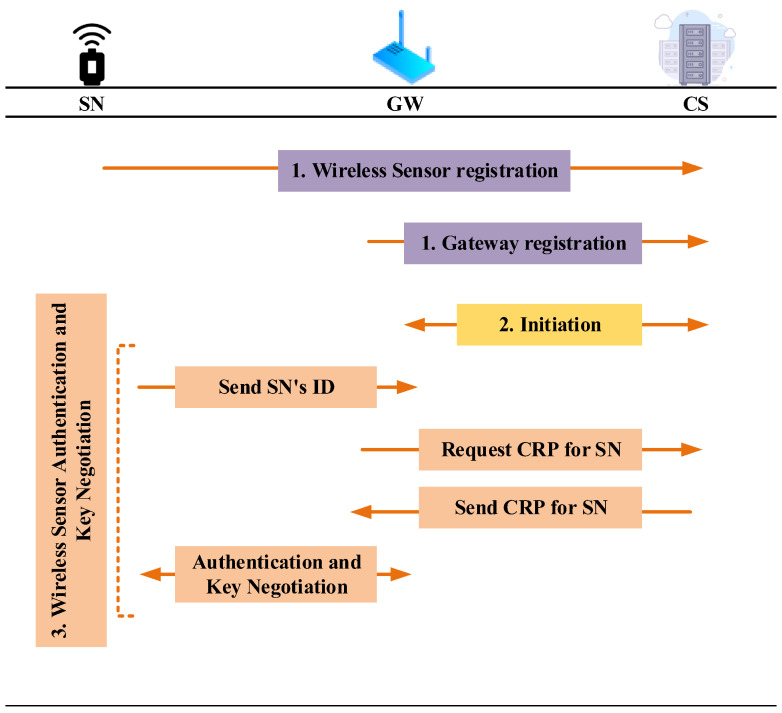
Protocol communication flowchart.

The existing wireless-sensor-authentication protocols do not consider the energy consumption of wireless sensors. Therefore, this paper proposes an efficient authentication protocol for IIoT-oriented wireless sensor networks. Firstly, wireless sensors and gateways are registered on the cloud server. Secondly, the gateway performs pre-authentication with the cloud server and saves the connection. Finally, the sensor sends its own identity information (challenge value) to the gateway. The gateway applies to the cloud server for the response value in the corresponding challenge–response pair. The gateway uses the response value to authenticate with the sensor. The protocol is divided into three phases: the registration phase, the initialization phase, and the wireless sensor authentication and key negotiation phase. Figure 2 provides a summary of the three phases.

### 4.1. Registration Stage

**Figure 3:**
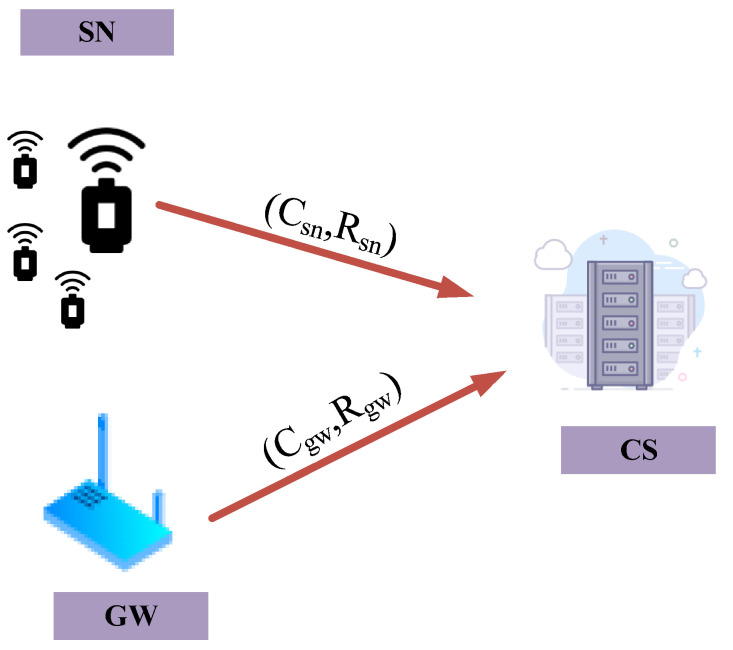
Registration process.

The wireless sensor generates a random challenge–response pair (CRP) during the registration process. It sends the CRP to the cloud server and retains the challenge value C in the CRP. After receiving the CRP, the cloud server maps the challenge value C in the CRP to an array of bits of length m through a k hash function in a Bloom filter. Additionally, the cloud server associates the response value R in the CRP. The registration process of the gateway is the same as the registration process of the wireless sensor. Figure 3 depicts the registration process between the wireless sensor and cloud server and the gateway and cloud server.

### 4.2. Initialization Stage

In the initialization phase, we adopted a pre-authentication mechanism. At this stage, the wireless sensor is dormant. We first perform authentication and key negotiation between the gateway and the cloud server. The initialization phase is gateway–cloud-server authentication and key negotiation. Algorithm 1 illustrates the specific process of the initialization communication flow phase. This phase is divided into the following steps.

**Step 1:** The gateway calculates $R_{gw}=PUF_{gw}\left(C_{gw}\right)$ through the PUF chip. The gateway then sends message $M_{1}=\left\{C_{gw}\right\}$ to the cloud server.

**Step 2:** After receiving message $M_{1}$, the cloud server maps $C_{gw}$ in the bit array of the Bloom filter and checks if there is a value of zero in the mapped position. If not, the associated response value is obtained by mapping the location $R_{gw}$. The cloud server selects a random number $N_{cs1}$, then calculates $X_{1}=NLF\left(R_{gw}\right)⊕N_{cs1}$, and then the cloud server sends message $M_{2}=\left\{H_{1},X_{1}\right\}$ to the gateway. The cloud server calculates the session key $SK_{1}=N_{cs1}\|R_{gw}$.

**Step 3:** After the gateway receives message $M_{2}$, it calculates $N_{cs1}^{\prime }=X_{1}⊕NLF\left(R_{gw}\right)$, $H_{1}^{\prime }=h\left(N_{cs1}^{\prime }\right)$, and compares it with the $H_{1}$ sent over. If $H_{1}^{\prime }\neq H_{1}$, it discards the message; otherwise, the gateway generates a new CRP and calculates the session key $SK_{1}=N_{cs1}^{\prime }\|R_{gw}$. Finally, the gateway computes $E_{1}={\left(C_{gwnew}\|R_{gwnew}\right)}_{Enc}^{SK_{1}}$, $H_{2}=h(C_{gwnew}\|R_{gwnew}\left\|N_{cs1}^{\prime }\right)$, and the gateway sends message $M_{3}=\left\{H_{2},E_{1}\right\}$ to the cloud server.

**Step 4:** After receiving message $M_{3}$, the cloud server calculates $D_{1}={\left(E_{1}\right)}_{Dec}^{SK_{1}}$, $H_{2}^{\prime }=h\left(D_{1}\|N_{cs1}\right)$, and compares it with the $H_{2}$ sent over. If $H_{2}^{\prime }=H_{2}$, the updated $C_{gwnew}$ and $R_{gwnew}$ can be obtained; otherwise, the message will be discarded. Finally, the cloud server sets the value of the $C_{gwnew}$ mapping location to 1 in the Bloom filter, while associating the corresponding $R_{gwnew}$.
**Algorithm 1** Initiation.GW: Calculate $R_{gw}=PUF_{gw}\left(C_{gw}\right)$           $GW\to CS:M_{1}=\left\{C_{gw}\right\}$CS:  Generate a random number $N_{cs1}$           Calculate $X_{1}=NLF\left(R_{gw}\right)⊕N_{cs1}$, $H_{1}=h\left(N_{cs1}\right)$           $CS\to GW:M_{2}=\left\{H_{1},X_{1}\right\}$           $SK_{1}=N_{cs1}\|R_{gw}$GW: Calculate $N_{cs1}^{\prime }=X_{1}⊕NLF\left(R_{gw}\right)$, $N_{cs1}^{\prime }=X_{1}⊕NLF\left(R_{gw}\right)$, $H_{1}^{\prime }=h\left(N_{cs1}^{\prime }\right)$           If $H_{1}^{\prime }\neq H_{1}$, the message is discarded. Otherwise, the gateway generates a new CRP.           $SK_{1}=N_{cs1}^{\prime }\|R_{gw}$           Calculate
$$E_{1}={\left(C_{gwnew}\|R_{gwnew}\right)}_{Enc}^{SK_{1}}$$
$$H_{2}=h(C_{gwnew}\|R_{gwnew}\left\|N_{cs1}^{\prime }\right)$$           $GW\to CS:M_{3}=\left\{H_{2},E_{1}\right\}$CS:  Calculate $D_{1}={\left(E_{1}\right)}_{Dec}^{SK_{1}}$, $H_{2}^{\prime }=h\left(D_{1}\|N_{cs1}\right)$           If $H_{2}^{\prime }=H_{2}$, we store the updated value $C_{gwnew}$ and $R_{gwnew}$.

### 4.3. Wireless Sensor Authentication and Key Negotiation Phase

The wireless sensor wakes up from hibernation mode every few minutes to collect environmental or device information. After data collection is complete, it will perform authentication and key negotiation with the gateway. It was mentioned in the assumptions that the gateway does not store any secrets about the wireless sensor. Thus, when the wireless sensor and the gateway perform authentication and key negotiation, the gateway needs to request the secrets of the relevant wireless sensor from the cloud server. This secret is used to complete the authentication and key negotiation between the gateway and the wireless sensor. Algorithm 2 illustrates the specific process of the wireless sensor communication process phase. This phase is divided into the following steps.

**Step 1:** The wireless sensor calculates $R_{sn}=PUF_{sn}\left(C_{sn}\right)$ by PUF chip. Then it selects a random number $N_{sn}$. After the wireless sensor divides $R_{sn}$ into $R_{sn1}$ and $R_{sn2}$, and it calculates $NLF(R_{sn1}⊕R_{sn2})$. Finally, the wireless sensor calculates $X_{2}=N_{sn}⊕NLF\left(R_{sn1}⊕R_{sn2}\right)$ and sends message $M_{4}=\left\{C_{sn},X_{2}\right\}$ to the gateway.

**Step 2:** After the gateway receives message $M_{4}$, it selects a random number $N_{gw}$ and calculates $H_{3}=h(C_{sn}\|N_{gw}\left\|SK_{1}\right)$. Then, it sends message $M_{5}=\left\{H_{3},C_{sn},N_{gw1}\right\}$ to the cloud server.

**Step 3:** After the cloud server receives message $M_{5}$, it calculates the message authentication code $H_{3}^{\prime }=h\left(C_{sn}\|SK_{1}\right)$. If $H_{3}^{\prime }=H_{3}$, the protocol proceeds to the next processing step; otherwise, the protocol discards the message. The cloud server then maps $C_{sn}$ into the Bloom filter’s bit array and checks the mapped position for a zero value. If not, the cloud server gets the associated response value by mapping the location $R_{sn}$. The cloud server divides $R_{sn}$ into $R_{sn1}$ and $R_{sn2}$, and it calculates $NLF(R_{sn1}⊕R_{sn2})$ and $NLF(R_{sn})$. The cloud server selects a random number $N_{cs2}$ and calculates $X_{3}=NLF\left(R_{sn}\right)⊕N_{cs2}$, $E_{2}={\left(NLF\left(R_{sn1}⊕R_{sn2}\right)\|X_{3}\right)}_{Enc}^{SK1}$, $H_{4}=h\left(NLF\left(R_{sn1}⊕R_{sn2}\right)\|X_{3}\right)$, $SK2=N_{cs2}⊕R_{sn}$. Finally, the cloud server sends message $M_{6}=\left\{H_{4},E_{2}\right\}$ to the gateway.

**Step 4:** After the gateway receives message $M_{6}$, it calculates $D_{2}={\left(E_{2}\right)}_{Dec}^{SK_{1}}$, $H_{4}^{\prime }=h\left(D_{2}\right)$. If $H_{4}^{\prime }\;\neq H_{4}$, the gateway discards the message. Otherwise, the gateway calculates $N{}_{sn}^{\prime }$$=NLF\left(R_{sn1}⊕R_{sn2}\right)⊕X_{2}$, $H_{5}=h\left(N{}_{sn}^{\prime }\|X_{3}\right)$. Finally, the gateway sends $M_{7}=\left\{X_{3},H_{5}\right\}$ to the corresponding wireless sensor.
**Algorithm 2** Wireless sensor authentication and key negotiation.SN: Calculate $R_{sn}=PUF_{sn}\left(C_{sn}\right)$           Generate a random number $N_{sn}$           Divides $R_{sn}$ into $R_{sn1}$ and $R_{sn2}$           Calculate $X_{2}=N_{sn}⊕NLF\left(R_{sn1}⊕R_{sn2}\right)$, $M_{4}=\left\{C_{sn},X_{2}\right\}$           $SN\to GW:M_{4}=\left\{C_{sn},X_{2}\right\}$GW: Generate a random number $N_{gw}$           Calculate $H_{3}=h(C_{sn}\|N_{gw}\left\|SK_{1}\right)$           $GW\to CS:M_{5}=\left\{H_{3},C_{sn},N_{gw}\right\}$CS:  Calculate $H_{3}^{\prime }=h(C_{sn}\|N_{gw}\left\|SK_{1}\right)$           If $H_{3}^{\prime }=H_{3}$, we look up the $C_{sn}$ and get the $R_{sn}$.           Divides $R_{sn}$ into $R_{sn1}$ and $R_{sn2}$           Calculate $NLF(R_{sn1}⊕R_{sn2})$           Generate a random number $N_{cs2}$           Calculate $X_{3}=NLF\left(R_{sn}\right)⊕N_{cs2}$           Calculate $SK2=N_{cs2}⊕R_{sn}$           $E_{2}={\left(NLF\left(R_{sn1}⊕R_{sn2}\right)\|X_{3}\right)}_{Enc}^{SK1}$           $H_{4}=h\left(NLF\left(R_{sn1}⊕R_{sn2}\right)\|X_{3}\right)$           $CS\to GW:M_{6}=\left\{H_{4},E_{2}\right\}$GW: Calculate $D_{2}={\left(E_{2}\right)}_{Dec}^{SK_{1}}$, $H_{4}^{\prime }=h\left(D_{2}\right)$           If $H_{4}^{\prime }\neq H_{4}$, discard the message; otherwise, count $N{}_{sn}^{\prime }=NLF\left(R_{sn1}⊕R_{sn2}\right)⊕X_{2}$.           Calculate $H_{5}=h\left(N{}_{sn}^{\prime }\|X_{3}\right)$           $GW\to SN:M_{7}=\left\{X_{3},H_{5}\right\}$SN: Calculate $H_{5}^{\prime }=h\left(N_{s}n\|X_{3}\right)$           If $H_{5}^{\prime }\neq H_{5}$, the message is discarded. Otherwise, wireless sensor generates a new CRP.           Calculate $D_{4}={\left(E_{4}\right)}_{Dec}^{R_{sn}}$, $N_{cs2}^{\prime }=R_{sn}⊕D_{4}$           $SK2=N{}_{cs2}^{\prime }⊕R_{sn}$, $E_{3}={\left(C_{snnew}\|R_{snnew}\right)}_{Enc}^{SK_{2}}$           $H_{6}=h(C_{snnew}\|R_{snnew}\left\|N{}_{cs2}^{\prime }\right)$           $SN\to GW:M_{8}=\left\{H_{6},E_{3}\right\}$GW: Forwards message $M_{8}=\left\{H_{6},E_{3}\right\}$ to the cloud serverCS:  Calculate $D_{3}={\left(E_{3}\right)}_{Dec}^{SK_{2}}$, $H_{6}^{\prime }=h\left(D_{4}\|N_{cs2}\right)$           if $H_{6}^{\prime }\neq H_{6}$, discard the message; otherwise, we store $C_{snnew}$ and $R_{snnew}$.

**Step 5:** After the wireless sensor receives message $M_{7}$, it calculates $H_{5}^{\prime }=h\left(N_{s}n\|X_{3}\right)$. If $H_{5}^{\prime }\neq H_{5}$, the wireless sensor discards the message. Otherwise, the wireless sensor generates a new CRP. The wireless sensor then computes $SK2=N{}_{cs2}^{\prime }⊕R_{sn}$, $E_{3}={\left(C_{snnew}\|R_{snnew}\right)}_{Enc}^{SK_{2}}$, $H_{6}=h(C_{snnew}\|R_{snnew}\left\|N{}_{cs2}^{\prime }\right)$. Additionally, the wireless sensor sends message $M_{8}=\left\{H_{6},E_{3}\right\}$ to gateway.

**Step 6:** After the gateway receives message $M_{8}$, it forwards message $M_{8}=\left\{H_{6},E_{3}\right\}$ to the cloud server.

**Step 7:** After the cloud server receives message $M_{8}$, it calculates $D_{3}={\left(E_{3}\right)}_{Dec}^{SK_{2}}$, $H_{6}^{\prime }=h\left(D_{4}\|N_{cs2}\right)$ according to the above method. If $H_{6}^{\prime }\neq H_{6}$, the cloud server discards the message. Otherwise, it updates the Bloom filter according to the above method.

In particular, the gateway and wireless sensor store the new and old challenge values during the initialization phase and the wireless-sensor-side authentication and key negotiation phase. A desynchronization attack on the protocol results in gateway–wireless-sensor and gateway–cloud-server authentication failure. The gateway and wireless sensors send $C_{old}$, and the cloud server looks for the $C_{old}$ in the Bloom filter. If it exists, the synchronization message is resent to the cloud server.

## 5. Security Evaluation

In this section, we perform an informal security analysis of the protocol and verify the protocol security using the AVISPA tool. Finally, we compare the security of our proposed protocol with other protocols.

### 5.1. Informal Security Analysis

Here, we elaborate on the protocol’s rich security features and use informal security analysis to demonstrate the protocol’s security.

**Mutual authentication:** The mutual authentication of gateway and cloud servers depends on challenge–response pairs and the validity of random numbers. The attacker cannot get the response value $R_{gw}$ in the challenge–response pair because he does not have access to the PUF chip of the gateway. The cloud server stores the challenge–response pairs generated in advance by the gateway. As a party participating in the communication, the gateway can authenticate the cloud server through $N_{cs1}^{\prime }$ and $H_{1}^{\prime }$. As a party participating in the communication, the cloud server can authenticate the gateway through $SK_{1}$ and $H_{2}^{\prime }$. The principle of gateway–wireless-sensor mutual authentication is the same as described above. Combined with the mutual authentication of wireless-sensor–gateway and gateway–cloud-server, the protocol indirectly realizes the mutual authentication of the wireless sensor-cloud server.

**Identity anonymity and un-traceability:** The protocol uses the challenge value $C_{sn}$ in the challenge–response pair as the wireless sensor identity and $C_{gw}$ as the gateway identity. However, the protocol updates the corresponding challenge–response pair after each successful authentication. Additionally, the identity information obtained by the attacker illegally changes randomly each time. Therefore, the attacker cannot determine the source of the information.

**Resist tampering attacks:** The attacker can intercept message $M_{1}\to M_{8}$ and can tamper with any of its values. The protocol guarantees message integrity by using hash functions. Specifically, in message $M_{1}$, the attacker tampering with $C_{gw}$ will cause the cloud server to fail to query in the Bloom filter. Additionally, the tampering with $X_{1}$ will cause message $M_{2}$ authentication to fail. Similarly, message $M_{4}$ is resistant to tampering attacks.

**Resist replay attacks:** The protocol uses a random number mechanism to prevent replay attacks. The random number varies from session to session. The attacker intercept a message in a session and replay this message in the next session, which will be discarded. Since the message contains a random number, the value of the random number has changed in this session. However, the value of the random number in the attacker’s replay message is an old value. Therefore, the replay attack is not valid for the protocol.

**Resist simulation and forgery attacks:** The attacker who wants to emulate legitimate sensors, gateways, and cloud servers or forge legitimate messages need to have the appropriate PUF chips to calculate the secret values $R_{sn}$ and $R_{gw}$ that can authenticate the identity. Since the attacker cannot obtain $R_{sn}$ and $R_{gw}$, it cannot forge a legitimate communication message to send to the gateway, sensors, and cloud servers. The attacker cannot properly decrypt these encrypted communication messages. Therefore, it is also impossible to obtain secret values to emulate legitimate sensors, gateways, and cloud servers.

**No clock synchronization:** The protocol design does not use a timestamp mechanism. This is because the use of the timestamp mechanism will cause clock synchronization problems [42,43]. The clock synchronization mechanism is not perfectly synchronized due to the latency problem of the network. The attacker can use the clock synchronization mechanism to attack the timestamp, which leads to a possible replay attack on the protocol.

**Resist physical attacks:** Wireless sensors are widely used in IIoT scenarios, and their distribution locations are scattered and unattended. Therefore, it is very vulnerable to physical attacks by the attacker. In the communication network model, we list wireless sensors and gateways as objects that are not physically protected. Thus, the attacker can capture wireless sensors and gateways and perform physical attacks on them to extract confidential information. In the protocol design, we introduce the PUF. Firstly, only the corresponding $C_{sn}$ and $C_{gw}$ are stored in the wireless sensors and gateways. $C_{sn}$ and $C_{gw}$ are transmitted as plaintext messages for communication and are not involved in the message encryption process as confidential. Thus, even if the attacker uses physical means to extract the *C* values of wireless sensors and gateways, he cannot achieve the purpose of the attack. Secondly, if the attacker tries to extract the PUF chip, the tiny physical changes in the PUF chip will be destroyed. Eventually, the PUF chip will be destroyed. The attacker cannot take out PUFs and embed them on their devices to simulate wireless sensors or gateways. To sum up, we believe that the use of PUFs in the protocol can ensure the security of the physical layer of the protocol.

**Forward and backward security of session keys:** Assuming that the attacker obtains the current session key, he cannot obtain the previous and subsequent session keys through the current session key. Additionally, he also cannot obtain the session key of the gateway–wireless-sensor through the session key of the cloud server-gateway. In this protocol, the session key is generated by random numbers and *R*. Thus there is no association between session keys, which ensures the forward and backward security of session keys.

**Resist internal attacks:** Suppose the attacker obtains all the secret values of one wireless sensor; he cannot obtain the secret values of other wireless sensors through this hijacked wireless sensor. Due to the introduction of PUFs in wireless sensors, the secret values generated by each wireless sensor are different. Additionally, the generation of session keys is also independent. Suppose the attacker captures the gateway, but the attacker cannot obtain the key SK2 between the wireless sensor and the cloud server, as we do not directly transmit the wireless sensor’s secret value $R_{sn}$ to the gateway. Thus, the protocol is resistant to internal attacks.

**Resisting desynchronization attacks:** A desynchronization attack on the protocol results in gateway–wireless-sensor and gateway–cloud-server authentication failure. The gateway and wireless sensors send $C_{old}$ and the cloud server looks for the $C_{old}$ in the Bloom filter. If it exists, the synchronization message is resent to the cloud server.

### 5.2. Validation of Automated Analysis Tools for Formal Security Protocols

**Figure 4:**
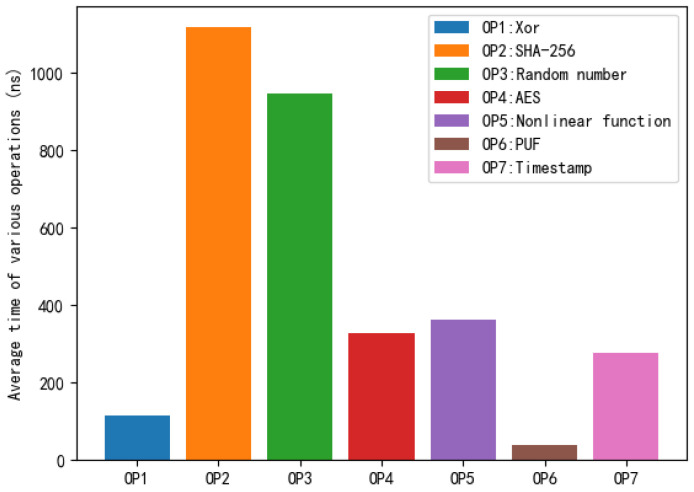
Average time taken by various operations.

In this subsection, we used the Automated Validation of Internet Security-Sensitive Protocols and Applications (AVISPA) tool to verify the protocol security. AVISPA is a toolset for automated certification of network security protocols and applications. It provides a modular expression language for specifying protocols and their security properties and integrates different backends implementing various state-of-the-art automated analysis techniques. The backend is divided into an on-the-fly model checker (OFMC), constrained logic-based attack searcher (CL-AtSe), SAT-based model checker (SATMC), and tree automata-based automatic approximate analysis security protocol (TA4SP). The protocol designer uses the High Level Protocol Specification Language (HLPSL) to emulate the protocol and specify the security target. The HLPSL2IF translator then converts the HLPSL specification to an IF specification, which is then fed into the AVISPA backend. Finally, AVISPA outputs the formal security analysis results [44]. We performed formal security analysis to verify the initialization phase, wireless sensor authentication, and key negotiation phases, respectively. The OFMC and CL-AtSe backends support XOR operations, so we used these two backends for formal security analysis verification. The left half of Figure 4 is the security analysis result of the initialization phase. The right half of Figure 4 is the security analysis result of the wireless sensor authentication and key agreement phase. We conducted a formal security analysis using a dynamic model checker backend and a constraint logic-based attack searcher backend for these two stages, respectively. Figure 4 shows that the two phases of the protocol are secure under the security analysis of the two backends.

### 5.3. Comparison of Security Features

Table 2 shows the comparison results of our proposed protocol with other protocols in terms of security features. In the protocol in [21], the gateway can obtain the secret value $R_{sn}$ of the wireless sensor. After the attacker captures the gateway, he can steal the communication information between the wireless sensor and the cloud server through SK1 and SK2. Therefore, the protocol in [21] is not resistant to insider attacks. At the same time, since the [21] protocol involves the update iteration of PUF, it has no synchronization mechanism. Therefore, the [21] protocol is vulnerable to desynchronization attacks. In the protocol in [45,46], there are two factors of account password and smart card. However, secrets in the smart card are easy to be stolen by the attacker, which reduces the security of the whole protocol. Therefore, they are vulnerable to physical attacks. The protocol in [46], uses timestamps to prevent replay attacks. Therefore, the protocol is vulnerable to timestamp attacks. The protocol in [47] stores secret values in the device, making it vulnerable to intrusive physical attacks.

## 6. Performance Evaluation

Reducing the computational cost and communication at the wireless sensor end is one of the main purposes of the protocol design. From the protocol level, the computing cost and communication cost of the wireless sensor end are reduced in a fine-grained way, so as to achieve the purpose of saving the energy consumption of the wireless sensor. In this section, we compare our protocol with other wireless-sensor-network authentication protocols in terms of wireless-sensor-side computation cost, wireless-sensor-side communication cost, total computational cost, and total communication cost. Table 3 shows the overall performance of the protocol. The comparison results are summarized in Table 3. Protocol performance comparison results: Firstly, in the protocol design, we introduce the PUF chip and we use the Bloom filter to save and query the challenge–response value generated by the PUF chip. It greatly reduces the total computational cost of wireless sensors and keeps the total communication cost of wireless sensors low. Combining these two aspects, our proposed protocol achieves the goal of reducing the energy consumption of the wireless sensor side at the authentication protocol level. Secondly, the protocol introduces a pre-authentication mechanism, and the gateway and the cloud server are continuously authenticated, which effectively reduces the total computing cost of the protocol. Finally, our proposed protocol performs poorly in terms of the total communication cost. This is due to the introduction of a pre-authentication mechanism, resulting in an increase in the number of messages. However, the communication cost on the wireless sensor side has always remained low. The increase in the total communication cost of the protocol lies in the increased communication cost between the gateway and the cloud server. In the industrial IoT scenario, the energy supply of gateways and cloud servers is sufficient, and the hardware conditions are relatively superior. Therefore, a small increase in the total communication cost of the protocol is acceptable. Next, we specifically carry out the performance evaluation of the protocol and related protocols in various aspects.

### 6.1. Comparison of Computational Costs of Wireless Sensors

**Figure 5:**
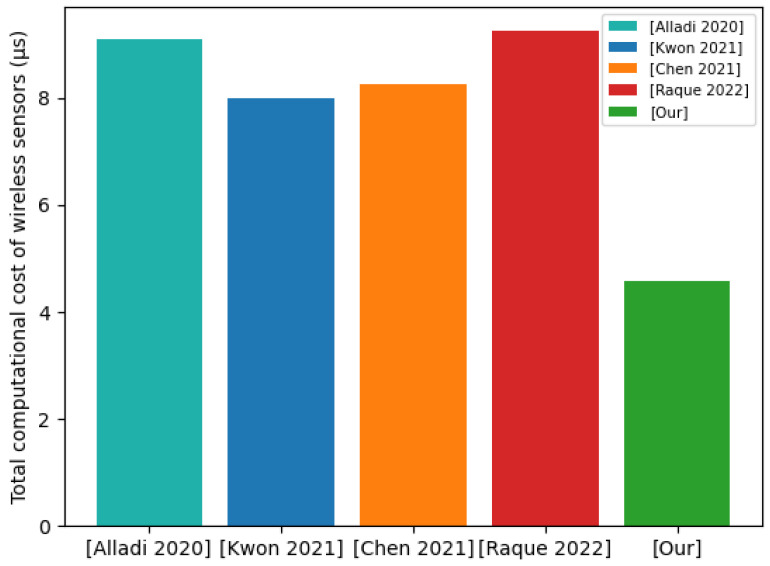
Total computational cost of wireless sensors.

Our proposed protocol uses various operations, including XOR, a hash algorithm (SHA-256), an AES symmetric encryption algorithm, a nonlinear function, a random number generation algorithm, and a PUF challenge–response pair. The protocol involves timestamp operations [46]. We used the MIRACL cryptographic library and the GMP (a large number library) in Microsoft Visual C++ software to implement operations such as XOR, timestamp, nonlinear function, the SHA-256 algorithm, the AES symmetric encryption algorithm, and random number generation for large numbers. Experimental environment: CPU processor: Intel(R) Core(TM) i5-5200U CPU @ 2.20 GHz, memory: 8.00 GB, OS: Win10 64-bit. The experimental environment and parameters are summarized in Table 4. The PUF chip takes about 40 ns to generate a 128-bit challenge–response pair [48]. We performed the above operations on the computer. In order to ensure accuracy, we performed the above operations 100,000 times, and took the average value as the last execution time. Figure 5 shows the average durations of various operations. We reserve three decimal places for the test results, and the final quantization results were as follows: Xor: 115.158 ns, SHA-256: 1117.060 ns, random number: 947.094 ns, AES: 326.310 ns, nonlinear function: 326.160 ns, PUF: 40 ns, and timestamp: 275.610 s. It is worth noting that most devices today have AES hardware acceleration. Therefore, the AES test speed here is the speed after AES hardware optimization.

When our proposed protocol is executed, the wireless sensor needs to send $M_{4}$ and $M_{8}$ messages and receive $M_{7}$ messages, which involve Xor, hashing, random number, PUF, AES, and non-linear operations. The specific quantities are as follows: $3T_{x}+2T_{h}+1T_{r}+2T_{p}+1T_{a}+2T_{f}$. When the [21] protocol is executed, the wireless sensor needs to perform the following operations: $15T_{x}+3T_{h}+2T_{r}+2T_{p}+1T_{a}+5T_{f}$. When the [45] protocol is executed, the wireless sensor needs to perform the following operations: $3T_{x}+6T_{h}+1T_{r}$. When the [46] protocol is executed, the wireless sensor needs to perform the following operations: $3T_{x}+6T_{h}+1T_{r}+1T_{t}$. When the [47] protocol is executed, the wireless sensor needs to perform the following operations: $2T_{x}+7T_{h}+2T_{a}+2T_{t}$.

**Figure 6:**
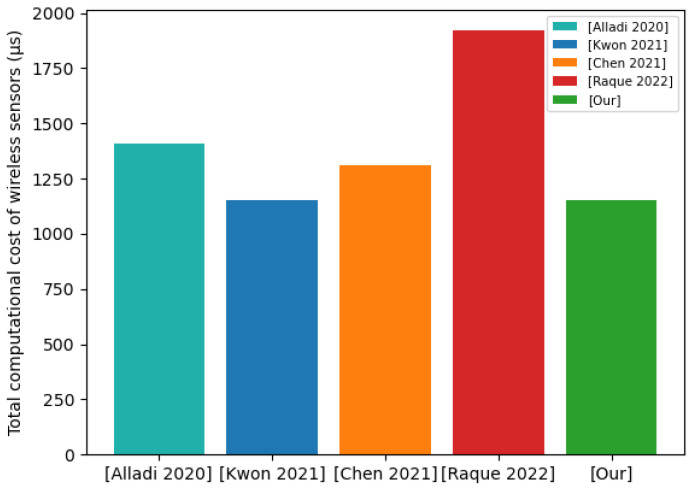
Communication cost of wireless sensors.

According to the average durations of various operations in Figure 5, we compared the total calculation costs of wireless sensors. Combined with the test results in Figure 5, we quantified the total computational costs on the wireless sensor side for the compared protocols. The total computational costs of the wireless sensor side of the [21] protocol, [45] protocol, [46] protocol, [47] protocol, and our proposed protocol were 9.110 μs, 7.995 μs, 8.271 μs, 9.252 μs, and 4.577 μs. Figure 6 shows the comparison results of the total computational costs of wireless sensors. In our protocol, the total computational costs of wireless sensors are significantly smaller than those of other protocols. Compared with the [21] protocol, we reduced the total computational costs of wireless sensors by 49.8%. Compared with the [45] protocol, we reduced the total computational costs of wireless sensors by 42.8%. Compared with the [46] protocol, we reduced the total computational costs of wireless sensors by 44.7%. Compared with the [47] protocol, we reduced the total computational costs of wireless sensors by 50.5%. It is worth noting that the [21] protocol has the same network communication model as our proposed protocol. Under the same communication model, we reduced the total computational cost on the wireless sensor side by 49.8%.

### 6.2. Comparison of the Communication Cost of Wireless Sensors

**Figure 7:**
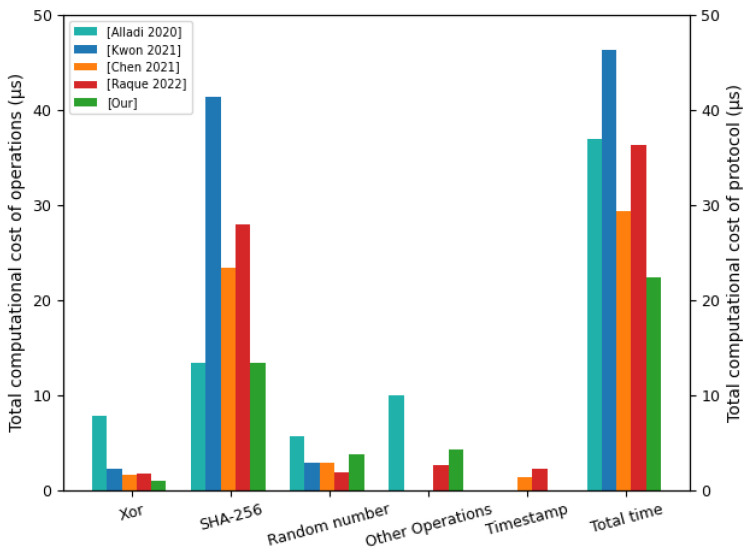
Total computational costs of operations and protocols.

In this subsection, we compare the communication costs on the wireless sensor side. In the communication subsystem of the wireless sensor, the wireless transceiver circuit has four states, namely, transmit, receive, idle, and sleep states. Among them, the energy consumption of sending and receiving is relatively high [49]. Therefore, we counted the messages sent and received by the wireless sensor. The operations involved in the protocol include: XOR operation, the hash algorithm (SHA-256), the AES symmetric encryption algorithm, a nonlinear function, a random number generation algorithm, a PUF challenge–response pair, and a timestamp. We assume that the AES algorithm outputs 256 bits, PUF generates 128 bits challenge–response pairs, the hash algorithm generates 256 bits summaries, and the random number algorithm generates 128 bit random numbers. On the wireless sensor side, our proposed protocol needs to send $M_{4}$ messages and $M_{8}$ messages, and receive $M_{7}$ messages. According to the analysis in the previous section, the communication cost of the $M_{4}=\left\{C_{sn},X_{2}\right\}$ message is 256 bits, the communication cost of the $M_{8}=\left\{H_{6},E_{3}\right\}$ message is 512 bits, and the communication cost of the $M_{7}=\left\{X_{3},H_{5}\right\}$ message is 384 bits. Therefore, the communication cost of our proposed protocol on the wireless sensor side is 256 + 512 + 384 = 1152 bits. We compared the communication cost with those of other protocols on the wireless sensor side. The communication cost of our proposed protocol on the wireless sensor side is 1152 bits. The communication cost of the [21] protocol on the wireless sensor side is 1408 bits. The communication cost of the [45] protocol on the wireless sensor side is 1152 bits. The communication cost of the [46] protocol on the wireless sensor side is 1312 bits. The communication cost of the [47] protocol on the wireless sensor side is 1920 bits. Figure 7 shows the comparison results of wireless sensor side communication costs. Our proposed protocol is the same as the [45] protocol in terms of the total communication cost on the wireless sensor side. However, it is better than [21,46,47] protocols in terms of total communication cost on the wireless sensor side.

### 6.3. Comparison of Total Computational Cost of Protocols

Our proposed protocol is an efficient authentication protocol for IIoT-oriented wireless sensor networks. In this subsection, we compare the total computational costs of protocols with other protocols. We calculated the total calculation cost of our proposed procotol and other protocols. Table 3 shows the numbers of operations required by the [21] protocol, the [45] protocol, the [46] protocol, the [47] protocol, and our proposed protocol.

When the [21] protocol is executed, the protocol needs to perform the following operations: (1) XOR; (2) hash algorithm; (3) random number; (4) PUF; (5) AES algorithm; (6) nonlinear function. The wireless sensor, gateway, and cloud server need to perform the following operations in total: $68T_{x}+12T_{h}+6T_{r}+4T_{p}+8T_{a}+20T_{f}$. When the [45] protocol is executed, the protocol needs to perform the following operations: (1) XOR; (2) hash algorithm; (3) random number. The wireless sensor, gateway, and user need to perform the following operations in total: $19T_{x}+37T_{h}+3T_{r}$. When the [46] protocol is executed, the protocol needs to perform the following operations: (1) XOR; (2) hash algorithm; (3) random number; (4) timestamp. The wireless sensor, gateway, and user need to perform the following operations in total: $14T_{x}+21T_{h}+3T_{r}+5T_{t}$. When the [47] protocol is executed, the protocol needs to perform the following operations: (1) XOR; (2) hash algorithm; (3) random number; (4) AES algorithm; (5) timestamp. The wireless sensor, gateway, and user need to perform the following operations in total: $15T_{x}+25T_{h}+2T_{r}+8T_{a}+8T_{t}$. When our proposed protocol is executed, the protocol needs to perform the following operations: (1) XOR; (2) hash algorithm; (3) random number; (4) PUF; (5) AES algorithm; (6) nonlinear function. The wireless sensor, gateway, and cloud server need to perform the following operations in total: $8T_{x}+12T_{h}+4T_{r}+4T_{p}+6T_{a}+6T_{f}$.

**Figure 8:**
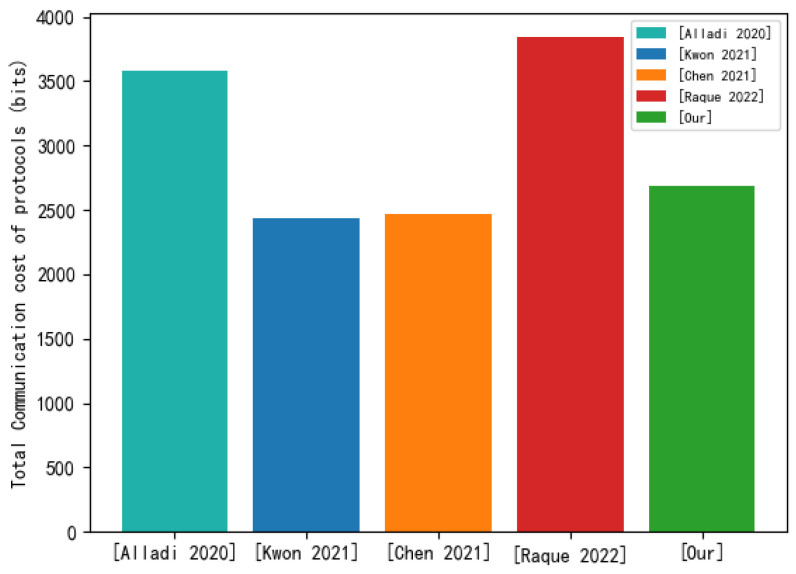
Total communication cost of protocols.

According to the average durations of various operations in Figure 5 and the number of operations required by the protocol in Table 5, we quantify the total computational costs of [21] protocol, [45] protocol, [46] protocol, [47] protocol, and our proposed protocol. We compared the total calculation costs of protocols and the total calculation costs of operations. The comparison results are shown in Figure 8. Figure 8 shows a comparison of the computational cost of various operations used by each protocol and the total computational cost of the protocols. Other operations include the AES symmetric encryption algorithm, nonlinear function, and PUF challenge–response pair. The total computational cost of the [21] protocol was 36.932 μs. The computational cost of the Xor operation was 7.831 μs. The computational cost of the SHA-256 operation was 13.405 μs. The computational cost of the random number operation was 5.683 μs. The computational cost of the other operations was 10.013 μs. The total computational cost of the [45] protocol was 46.360 μs. The computational cost of the Xor operation was 2.188 μs. The computational cost of the SHA-256 operation was 41.331 μs. The computational cost of the random number operation was 2.841 μs. The total computational cost of the [46] protocol was 29.289 μs. The computational cost of the Xor operation was 1.612 μs. The computational cost of the SHA-256 operation was 23.458 μs. The computational cost of the random number operation was 2.841 μs. The computational cost of the timestamp operation was 4.291 μs. The total computational cost of the [47] protocol was 36.363 μs. The computational cost of the Xor operation was 1.727 μs. The computational cost of the SHA-256 operation was 27.927 μs. The computational cost of the random number operation was 1.894 μs. The computational cost of the other operations was 2.610 μs. The computational cost of the timestamp operation was 2.205 μs. The total computational cost of the our proposed protocol was 22.404 μs. The computational cost of the Xor operation was 0.921 μs. The computational cost of the SHA-256 operation was 13.404 μs. The computational cost of the random number operation was 3.788 μs. The computational cost of the other operations was 4.291 μs. From Figure 8, we can clearly see that our proposed protocol has an advantage in the total computational costs.

### 6.4. Comparison of Total Communication Costs of Protocols

The communication cost analysis of our proposed protocol is as follows: We use the pre authentication mechanism to complete the mutual authentication and key negotiation between the gateway and the cloud server in the initialization stage. A large number of wireless sensors need to carry out mutual authentication and key negotiation with cloud servers, but in this process, the mutual authentication and key negotiation between gateway and cloud services only needs to be performed once. Therefore, the communication cost incurred in the protocol initialization phase can be ignored. When the proposed protocol is executed, $M_{4}$–$M_{8}$ messages need to be transmitted. The sensor sends a message $M_{4}=\left\{C_{sn},X_{2}\right\}$ to the gateway. $C_{sn}$ is the challenge value of 128 bits in the challenge–response pair, and $X_{2}$ is a 128 bit XOR value. Therefore, the communication cost of an $M_{4}$ message is 128 + 128 = 256 bits. The gateway sends an $M_{5}=\left\{H_{3},C_{sn},N_{gw}\right\}$ message to the cloud server. $H_{3}$ is a 256-bit hash value, $C_{sn}$ is a 128-bit challenge value in a challenge–response pair, and $N_{gw}$ is a 128-bit random number. Therefore, the communication cost of $M_{5}$ message is 256 + 128 + 128 = 512 bits. The cloud server sends an $M_{6}=\left\{H_{4},E_{2}\right\}$ message to the gateway. $H_{4}$ is a 256-bit hash value, and $E_{2}$ is a 256-bit AES encrypted value. Therefore, the communication cost of an $M_{6}$ message is 256 + 256 = 512 bits. The gateway sends an $M_{7}=\left\{X_{3},H_{5}\right\}$ message to the sensor. $X_{3}$ is a 128-bit XOR value, and $H_{3}$ is a 256-bit hash value. Therefore, the communication cost of an $M_{7}$ message is 128 + 256 = 384 bits. The sensor sends an $M_{8}=\left\{H_{6},E_{3}\right\}$ message to the gateway. $H_{6}$ is a 256-bit hash value, and $E_{3}$ is a 256-bit AES encrypted value. Therefore, the communication cost of an $M_{8}$ message is 256 + 256 = 512 bits. The gateway also needs to forward the $M_{8}$ message to the cloud server. To sum up, the total communication cost of our proposed protocol is 256 + 512 + 512 + 384 + 512 + 512 = 2688 bits.

We have statistics of communication costs for each protocol. The total communication cost of the [21] protocol is 3584 bits. The total communication cost of the [45] protocol is 2432 bits. The total communication cost of the [46] protocol is 2496 bits. The total communication cost of the [47] protocol is 3840 bits. The total communication cost of our proposed protocol is 2688 bits. Figure 8 shows the total communication cost comparison between our proposed protocol and other protocols. From the comparison results, the total communication cost of our proposed protocol is better than those of the [21] protocol and the [47] protocol. The total communication cost of our proposed protocol is slightly higher than those of the [45,46] protocols. In general, industrial gateways and cloud servers have continuous power supplies. The throughput of their network cards is large, and they support duplex mode. Therefore, from the perspective of energy consumption and throughput, the total communication cost of the proposed protocol is acceptable.

## 7. Conclusions

This paper proposes an efficient authentication protocol for IIoT-oriented wireless sensor networks. The purpose is to reduce the energy consumption of wireless sensors in a fine-grained manner at the protocol level. The protocol introduces PUF chips and uses Bloom filters to save and query challenge–response pairs generated by PUF chips. While ensuring the physical security of the device, the computing cost and communication cost of the wireless sensor are reduced. The protocol introduces a pre-authentication mechanism, which reduces the overall computing cost of the protocol. Finally, detailed comparative experiments demonstrate that our proposed protocol has more security properties. The protocol significantly reduces the computational cost and communication cost on the wireless sensor side, and the total computational cost of the protocol. We believe this protocol will be well suited for wireless-sensor-network authentication in IIoT scenarios.

On a separate regard, although we reduce the total computational cost of wireless sensor terminals and protocols, the total communication cost of protocols is controlled within acceptable limits. The protocol increases communication costs between industrial gateways and cloud servers. In order to further optimize our proposed protocol, we will be prepared to reduce the communication cost of the industrial gateway and cloud server. In addition, we consider using new security primitives to design authentication protocols.

## Figures and Tables

**Table 1:** Symbol meaning table.

Notation	Description
$C_{gw},C_{sn},C_{snnew},C_{gwnew}$	Challenge value for the challenge-response pair
$PUF_{gw},PUF_{sn}$	Physically unclonable functions
$R_{gw},R_{sn},R_{snnew},R_{gwnew}$	Response value of the challenge-response pair
$N_{sn},N_{gw},N_{cs1},N_{cs2}$	Random number
*H*	Hash value
h()	Hash algorithm
⊕,‖	Xor operation and join operation
*M*	Communication message
${\left(\right)}_{Enc}^{SK},{\left(\right)}_{Dec}^{SK}$	Symmetric encryption and Symmetric decryption
E,D	Encrypted and Decrypted values
NLF()	Nonlinear function

**Table 2:** Comparison of security features table.

Protocol	ST1	ST2	ST3	ST4	ST5	ST6	ST7	ST8	ST9	ST10
Alladi 2020 [21]	🗸	🗸	🗸	🗸	🗸	🗸	✕	🗸	🗸	✕
Kwon 2021 [45]	🗸	🗸	🗸	🗸	🗸	🗸	✕	🗸	🗸	🗸
Chen 2021 [46]	🗸	🗸	🗸	🗸	🗸	✕	✕	🗸	🗸	🗸
Raque 2022 [47]	🗸	🗸	🗸	🗸	🗸	🗸	✕	🗸	🗸	🗸
Our	🗸	🗸	🗸	🗸	🗸	🗸	🗸	🗸	🗸	🗸

ST1: Mutual authentication; ST2: Identity anonymity and untraceability; ST3: Resist tampering attacks; ST4: Resist repaly attacks; ST5: Resist simulation and forgery attacks; ST6: No clock synchronization; ST7: Resist physical attacks; ST8: Forward and backward security of session keys; ST9: Resist internal attacks; ST10: Resist desynchronization attacks.

**Table 3:** Overall performance of the protocol.

Protocol	Total Computational Cost of Wireless Sensors	Total Communication Cost of Wireless Sensors	Total Computational Cost of Protocols	Total Communication Cost of Protocols
Alladi 2020 [21]	9.110 μs	1408 bits	36.932 μs	3584 bits
Kwon 2021 [45]	7.995 μs	1152 bits	46.360 μs	2432 bits
Chen 2021 [46]	8.271 μs	1312 bits	29.289 μs	2496 bits
Raque 2022 [47]	9.252 μs	1920 bits	36.363 μs	3840 bits
Our	4.577 μs	1152 bits	22.404 μs	2688 bits

**Table 4:** The experimental environment and parameters.

CPU Processor	Memory	OS	Software Tools	Toolset
Intel(R) Core(TM) i5-5200U CPU @ 2.20 GHz	8.00 GB	Win10 64-bit	Microsoft Visual C++	MIRACL cryptographic library and GMP large number library

**Table 5:** The number of operations.

Protocol	Sensor Node	Gateway	Cloud Server/User	Total
Alladi 2020 [21]	$15T_{x}+T_{h}+2T_{r}+2T_{p}+1T_{a}+5T_{f}\approx 9.110\;\mu s$	$30T_{x}+4T_{h}+3T_{r}+2T_{p}+3T_{a}+9T_{f}\approx 15.003\;\mu s$	$23T_{x}+5T_{h}+1T_{r}+4T_{a}+6T_{f}\approx 12.659\;\mu s$	$68T_{x}+12T_{h}+6T_{r}+4T_{p}+8T_{a}+20T_{f}\approx 36.932\;\mu s$
Kwon 2021 [45]	$3T_{x}+6T_{h}+1T_{r}\approx 7.995\;\mu s$	$9T_{x}+18T_{h}+1T_{r}\approx 22.091\;\mu s$	$7T_{x}+13T_{h}+1T_{r}\approx 16.275\;\mu s$	$19T_{x}+37T_{h}+3T_{r}\approx 46.360\;\mu s$
Chen 2021 [46]	$3T_{x}+6T_{h}+1T_{r}+1T_{t}\approx 8.271\;\mu s$	$6T_{x}+7T_{h}+1T_{r}+2T_{t}\approx 10.009\;\mu s$	$5T_{x}+8T_{h}+1T_{r}+2T_{t}\approx 11.011\;\mu s$	$14T_{x}+21T_{h}+3T_{r}+5T_{t}\approx 29.289\;\mu s$
Raque 2022 [47]	$2T_{x}+7T_{h}+2T_{a}+2T_{t}\approx 9.252\;\mu s$	$4T_{x}+8T_{h}+1T_{r}+4T_{a}+4T_{t}\approx 12.752\;\mu s$	$9T_{x}+10T_{h}+1T_{r}+2T_{a}+2T_{t}\approx 14.358\;\mu s$	$15T_{x}+25T_{h}+2T_{r}+8T_{a}+8T_{t}\approx 36.363\;\mu s$
Our	$3T_{x}+2T_{h}+1T_{r}+2T_{p}+1T_{a}+2T_{f}\approx 4.577\;\mu s$	$2T_{x}+5T_{h}+1T_{r}+2T_{p}+2T_{a}+1T_{f}\approx 7.778\;\mu s$	$3T_{x}+5T_{h}+2T_{r}+3T_{a}+3T_{f}\approx 9.890\;\mu s$	$8T_{x}+12T_{h}+4T_{r}+4T_{p}+6T_{a}+6T_{f}\approx 22.404\;\mu s$

$T_{x}:\mathrm{Xor},T_{h}:\mathrm{Hash},T_{r}:\mathrm{Random}\;\mathrm{number},T_{p}:\mathrm{PUF},T_{a}:\mathrm{AES},T_{f}:\mathrm{Nonlinear}\;\mathrm{function},T_{t}:\mathrm{Time}\;\mathrm{stamp}$.

## Data Availability

Not applicable.
